# Is automatic cephalometric software using artificial intelligence better than orthodontist experts in landmark identification?

**DOI:** 10.1186/s12903-023-03188-4

**Published:** 2023-07-08

**Authors:** Huayu Ye, Zixuan Cheng, Nicha Ungvijanpunya, Wenjing Chen, Li Cao, Yongchao Gou

**Affiliations:** 1 Department of Orthodontics, Stomatological Hospital of Chongqing Medical University, 426#, Songshi North Road, Yubei District, Chongqing, 401147 PR China; 2 Chongqing Key Laboratory of Oral Diseases and Biomedical Sciences, 426#, Songshi North Road, Yubei District, Chongqing, 401147 PR China; 3 Chongqing Municipal Key Laboratory of Oral Biomedical Engineering of Higher Education, 426#, Songshi North Road, Yubei District, Chongqing, 401147 PR China; 4 Chongqing Haochi Private Dental Clinic, No. 711, Konggang Avenue, Yubei District, Chongqing, 401147 PR China; 5 Faculty of Dentistry, Chulalongkorn University, 34 Henri Dunant Road, Pathumwan, Bangkok, 10330 Thailand

**Keywords:** Cephalometric tracings, Artificial intelligence, Automatic digitization

## Abstract

**Background:**

To evaluate the techniques used for the automatic digitization of cephalograms using artificial intelligence algorithms, highlighting the strengths and weaknesses of each one and reviewing the percentage of success in localizing each cephalometric point.

**Methods:**

Lateral cephalograms were digitized and traced by three calibrated senior orthodontic residents with or without artificial intelligence (AI) assistance. The same radiographs of 43 patients were uploaded to AI-based machine learning programs MyOrthoX, Angelalign, and Digident. Image J was used to extract x- and y-coordinates for 32 cephalometric points: 11 soft tissue landmarks and 21 hard tissue landmarks. The mean radical errors (MRE) were assessed radical to the threshold of 1.0 mm,1.5 mm, and 2 mm to compare the successful detection rate (SDR). One-way ANOVA analysis at a significance level of *P* < .05 was used to compare MRE and SDR. The SPSS (IBM-vs. 27.0) and PRISM (GraphPad-vs.8.0.2) software were used for the data analysis.

**Results:**

Experimental results showed that three methods were able to achieve detection rates greater than 85% using the 2 mm precision threshold, which is the acceptable range in clinical practice. The Angelalign group even achieved a detection rate greater than 78.08% using the 1.0 mm threshold. A marked difference in time was found between the AI-assisted group and the manual group due to heterogeneity in the performance of techniques to detect the same landmark.

**Conclusions:**

AI assistance may increase efficiency without compromising accuracy with cephalometric tracings in routine clinical practice and research settings.

## Introduction

Early in 1931, Broadbent and Hofrath introduced lateral cephalometric radiographs, setting a precedent for their application in orthodontics practice and research. Since then, conventional cephalometry has become a standardized diagnostic method for malocclusion analysis and treatment planning [1]. Based on the identification of anatomical landmarks, cephalometric analysis is conducted on angles and distances measurement for the interpretation of craniofacial structures. Cephalometric analysis was initially coined to manually localize landmarks on acetate overlays over a lighted view box and measure the linear and angular values with a protractor, which is tedious, time-consuming, and subjective [2].

Defined by John McCarthy in 1956, AI now serves as a branch of computer science that has been receiving the spotlight and is now widely used in different fields, especially in biological and medical diagnostics. AI-derived machine-learning approaches have been developed by imitating biological networks through computer programs that model the way the intelligent human performs [3]. Since the radiographs represent biological shapes, they cannot be described in terms of shifted and rotated patterns that could be easily recognized. Recent years have witnessed the advances and integration of AI in medicine [4, 5], which is driven by the development of deep learning algorithms, computing hardware advances, and the exponential growth of data. The application of AI currently gained wide attention for a plethora of medical purposes, especially for decision-making and recognition of objects.

In orthodontics, cephalometric analysis with the assistance of AI is applied to the evaluation of post-treatment results and prediction of growth [6–8]. The evolution from manual cephalometric analysis to AI-assisted cephalometric analysis is aimed at improving the diagnostic value by reducing measurement errors and saving clinical time [9–11]. As multidimensional data is increasingly being generated in routine care, AI can support clinicians to reach consistency in diagnosis and treatment. Through the YOLOv3 method, Hwang et al. evaluated 283 lateral cephalometric images with 46 hard tissue and 32 soft tissue landmarks and found that the mean detection error between AI and orthodontists was 1.46 ± 2.97 mm [12, 13]. Similar to their results, Kim et al. found that the overall automated detection error of landmarks identification using cascaded convolutional neural networks was 1.55 ± 2.17 mm [14]. However, some researchers announced that the AI-assisted cephalometric analysis was not reproducible due to large inter- and intra-variability errors in landmark annotation [15]. By applying a statistical simulation procedure, Moon et al. proposed that the accuracy of AI was directly proportional to the quantity of learning data and the number of detection targets. Considering the inter-examiner difference, a sufficient quantity of learning data sets (approximately at least 2300) was necessary to develop accurate AI [16].

Many commercially available software developed in cephalometry; however, the software algorithms developed did not seem accurate enough in clinical practice [17]. AI-assisted detection has been identified as useful since landmark identification is a laborious task, requiring the time of experienced experts [18]. However, the assessment of common commercial software and the impact of experienced orthodontists-AI collaboration on the accuracy of cephalometric landmark detection are lacking. Among the commercial software, current AI-assisted programs (MyOrthoX, Angelalign, and Digident) outperformed state-of-the-art landmark identification methods, providing process automation through a knowledge-based algorithm. The present study aimed to evaluate and compare the accuracy of manually traced lateral cephalograms with automatic, or AI-assisted programs, allowing orthodontists to make an informed choice of suitable software and analysis methods.

## Material and methods

### Trial design

All procedures performed were in accordance with the ethical standards of the Clinical Research Ethics Committee of Chongqing Medical University (Approval No.2022–077). In this study, a total of 43 samples were collected from ethnic groups in the southern and southeast parts of China. The patient’s radiographs with the same resolution and quality were randomly collected from Chongqing Medical University. Patient data were handled according to the CONSORT (Consolidated Standards of Reporting Trials) Statement and Helsinki Declaration. The radiographs belonged to those who had undergone orthodontic treatment between June 2018 and May 2022. The following were the inclusion criteria: no cleft lip and palate, and no diagnosed systemic diseases or craniofacial syndromes. In the survey, the patients and their parents or guardians were informed about the aim of the study, the privacy policy, and their right to refuse to participate.

### Identification of cephalometric landmarks

**Fig. 1:**
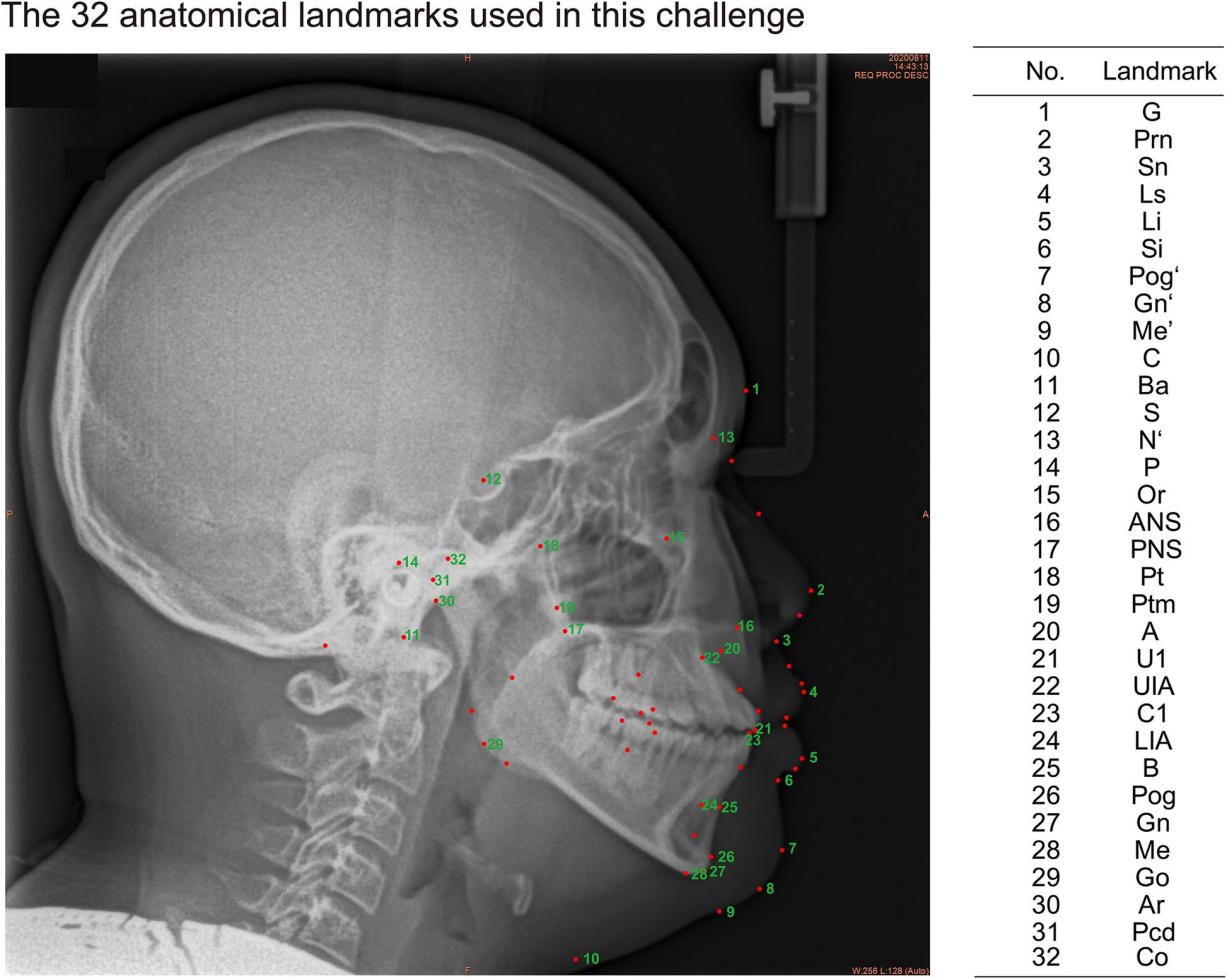
The 32 anatomical landmarks used in this challenge. All landmarks are defined and explained in Table 1

Thirty-two commonly used skeletal and dental cephalometric points were selected including 21 hard tissue and 11 soft tissue landmarks (Fig. 1). Two coordinate fiducials were marked on the radiographs to construct a reference grid, and the line connecting these two points is the z-axis [19]. The beginning and end of the 30-mm virtual ruler in AI were manually aligned with the ruler on radiographs to allow comparison. Each landmark’s definition, position, and abbreviation were described in Table 1.

**Table 1:** List of anatomical landmarks used

Landmark number	Explanation	Abbreviation
1	Glabella	G
2	Pronasale	Prn
3	Subnasale	Sn
4	Labrale superius	Ls
5	Labrale inferius	Li
6	Mentolabial sulcus	Si
7	Pogonion of soft tissue	Pog ‘
8	Gnathion of soft tissue	Gn ‘
9	Menton of soft tissue	Me ‘
10	Cervical point	C
11	Basion	Ba
12	Sella	S
13	Nasion	N
14	Porion	P
15	Orbitale	Or
16	Anterior nasal spine	ANS
17	Posterior nasal spine	PNS
18	Pterygoid	Pt
19	Pterygomaxillary fissure	Ptm
20	Subspinale	A
21	Upper incisor	U1
22	Root apex of upper central incisor	UIA
23	Lower incisor	C1
24	Root apex of lower central incisor	LIA
25	Supramental	B
26	Pogoion	Pog
27	Gnathion	Gn
28	Menton	Me
29	Gonion	Go
30	Articular	Ar
31	Posterior condyle	Pcd
32	Condylion	Co

For the manual group, all landmarks were manually digitized by a single observer and confirmed by other two observers with any discrepancies adjudicated by mutual agreement. Before registration, three observers, all staff members of the Orthodontic Department, calibrated with respect to the definition of the landmarks. The 33 radiographs were coded and presented to the observers in random order. Landmark identification was performed manually using a mouse-controlled cursor.

**Fig. 2:**
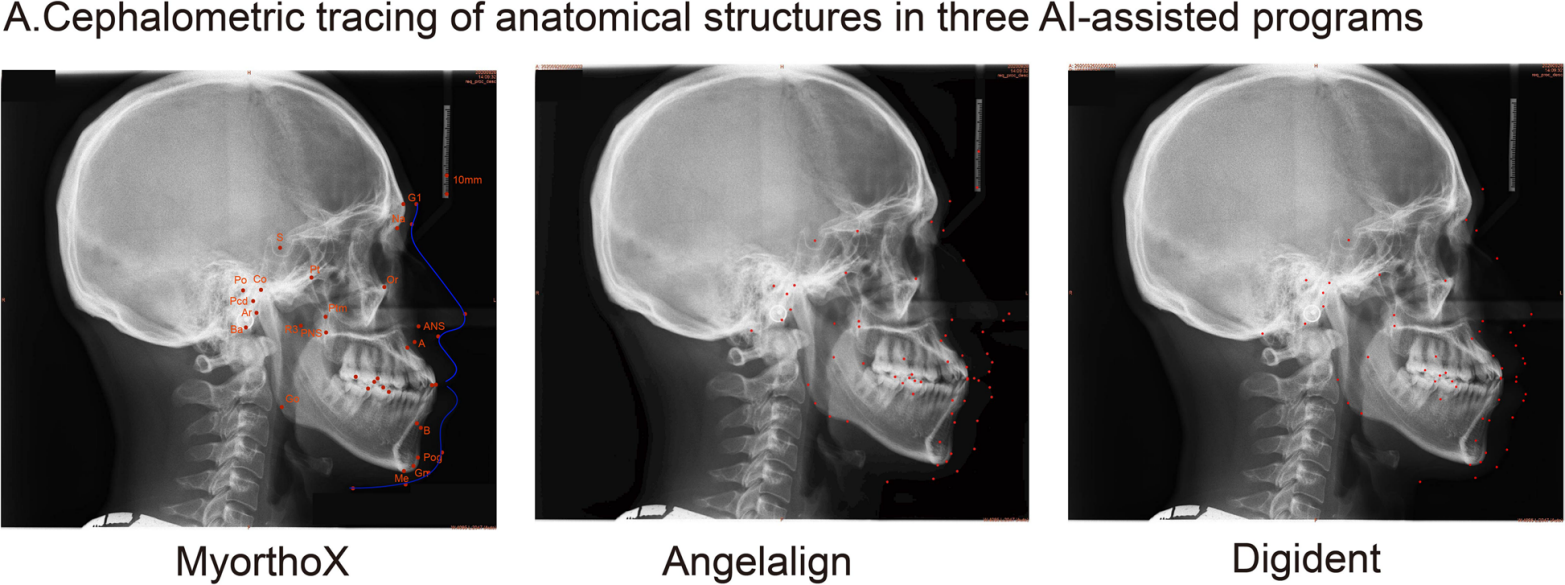
Cephalometric tracing of anatomical structures in three AI-assisted programs. Sample lateral cephalometric radiograph with a 30-mm ruler uploaded to the MyOrthoX, Angelalign, and Digident programs

For the automated detection group, the cephalograms were uploaded to three commercial software (MyOrthoX, Angelalign, and Digident) with no further labeling or changes, and the landmarks were auto-identified (Fig. 2). Tracings of the manual groups and automated detection groups were then scanned into Image J to obtain the coordinates of each landmark.

For the AI-assisted group, landmarks were digitized with orthodontists after the automated landmark identification using the software. To prevent the recognition of previous landmarks, all markings were removed and the landmark locating was conducted after 2 weeks. Furthermore, to determine the intra-observer error, all examiners conducted a second digitization of the cephalograms after a 2-week interval to provide the ground truth for evaluating the value of AI assistance.

### Evaluation matrices

Two main criteria are considered to evaluate the performance of submitted methods. The positions of the landmarks were identified using the x- and y- coordinates. Distance error (DE) was defined as the Euclidean distance between the manually annotated landmark coordinates and estimated landmark coordinates by AI. xi, yi denote the coordinates from MyOrthoX, Angelalign, and Digident; × 1, y1 denote coordinates from the orthodontist group.

$$DE=\sqrt{{(xi-x1)}^{2}+{(yi-y1)}^{2}} (mm)$$

The MRE and SD for each landmark (i) were calculated by the equations below, where n was the number of test images.

$$MRE_i\;=\;\frac{\sum_{j=1}^n\;DE_i}n\;\left(mm\right)\;\;\;\;\;\;\;SD_i=\sqrt{\frac{\sum_{j=1}^n\;\left(DE_i\;-\;MDE_i\right)}n\;\left(mm\right)}$$

Followed by the format of previous accuracy reports, thereby making the analogous comparison, the successful detection ratio (SDR) for 1.0-, 1.5-, and 2.0-mm ranges were calculated for 32 landmarks. Mathematically, SDR can be defined as follows:

$$SDR=\frac{number\;of\;sucessfully\;detected\;landmarks\;with\;respect\;to\;p}N$$

In this equation, p means the precision ranges of 1.0, 1.5, and 2.0 mm. N denotes the sample capacity.

### Time analysis

The average time needed for each group was measured in seconds using a stopwatch. For the manual group, the analyzing time included the process of locating the landmarks and access to skeletal and dental anatomical structures, performed by three orthodontists. For the automated detection group, the time the programs took to identify the anatomical points and present the data sets in different analytic approaches was recorded. For the AI-assisted group, the analyzing time included plotting the landmarks by one observer as measurements of angles and distances were automatically calculated by the AI programs.

### Statistical analysis

Three automatic landmark-detection software were analyzed for each landmark. One-way ANOVA analysis was applied to compare the average SDR among MyOrthoX, Angelalign, Digident, and orthodontist groups within 1.0, 1.5, and 2.0 mm thresholds. Paired t-test was conducted to compare the average time for cephalometric analysis between AI-assistant groups and manual groups. All data were analyzed using SPSS Statistics (version 27; IBM Corp., Armonk, NY, USA) and PRISM (version 8.0.2; GraphPad Software, Inc.; San Diego, CA, USA). 95% confidence intervals are given with statistical significance set at *p* < 0.05.

## Results

### Comparison of the three automated AI-based landmark detection programs

#### MRE

**Fig. 3:**
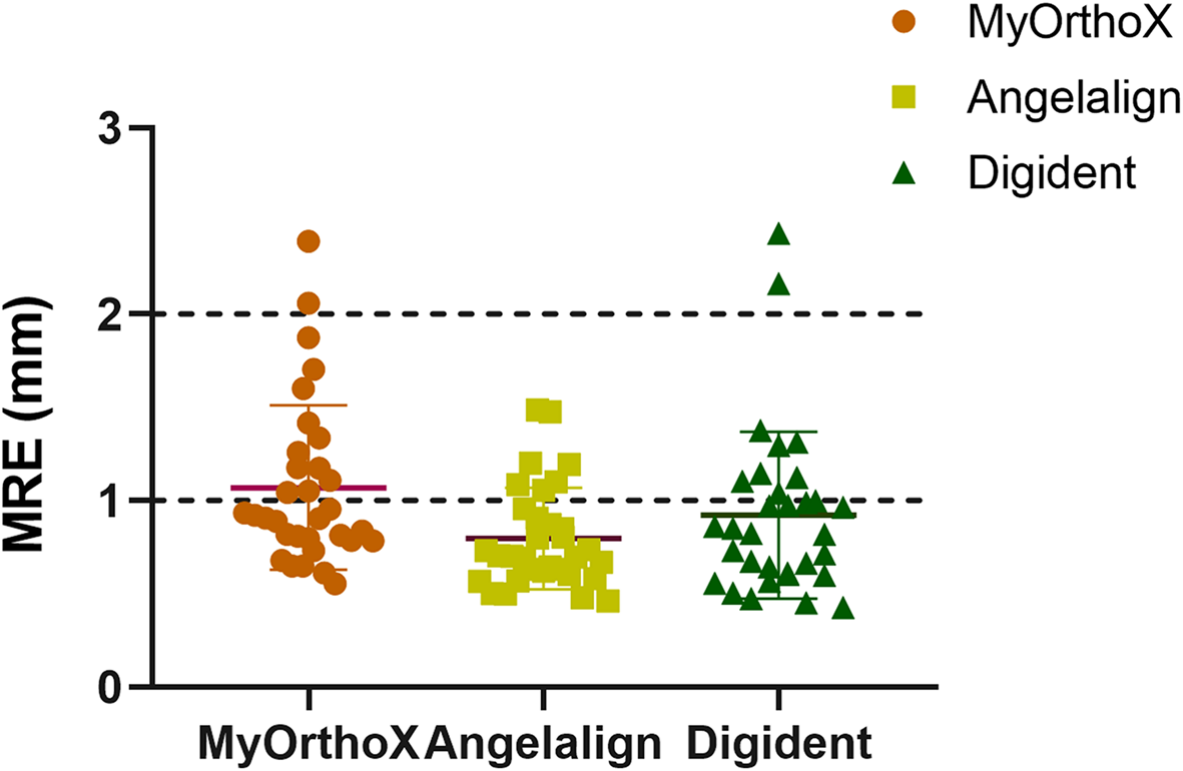
Mean radical error (MRE) for each landmark measured by three AI-assisted programs

**Fig. 4:**
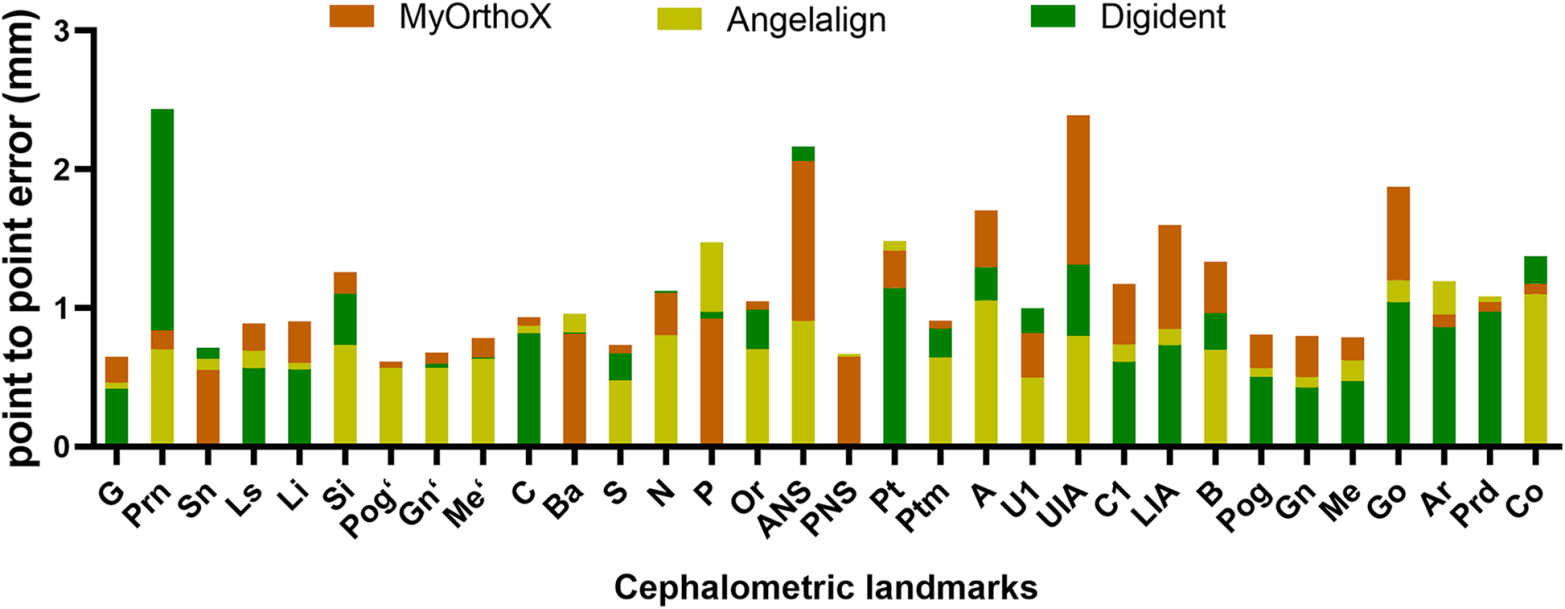
Mean radical error (MRE) for all Landmarks measured by three AI-assisted programs. One-way ANOVA analysis was applied to compare the MRE among the groups

The Angelalign dataset showed the lowest average MRE of 0.80 ± 0.26 mm, while Digident and MyOrthoX showed an average MRE of 1.11 ± 0.48 mm and 0.97 ± 0.51 mm, respectively (Fig. 3). Among the 32 landmarks, the glabella (G) exhibited the lowest MRE (0.52 ± 0.46 mm), while the anterior nasal spine (ANS) exhibited the highest MRE (1.71 ± 1.29 mm). A detailed comparison between MyOrthoX, Angelalign, and Digident in terms of the MRE was shown in Fig. 4 and Table 2.

**Table 2:** Mean relative error (MRE) and standard derivation (SD) for the 32 Landmarks measured by each software

Landmark	MRE ± SD (mm)			
	MyOrthoX	Angelalign	Digident	Average
G	0.65 ± 0.42	0.46 ± 0.54	0.45 ± 0.37	0.52 ± 0.46
Prn	0.84 ± 0.43	0.70 ± 1.21	2.43 ± 1.57	1.32 ± 1.41
Sn	0.55 ± 0.32	0.63 ± 0.71	0.71 ± 0.39	0.63 ± 0.51
Ls	0.89 ± 0.66	0.69 ± 0.83	0.57 ± 0.33	0.71 ± 0.66
Li	0.90 ± 0.49	0.60 ± 0.63	0.56 ± 0.29	0.69 ± 0.52
Si	1.26 ± 0.68	0.73 ± 0.68	1.10 ± 0.65	1.03 ± 0.71
Pog’	0.61 ± 0.35	0.58 ± 0.72	0.60 ± 0.37	0.60 ± 0.51
Gn’	0.68 ± 0.39	0.57 ± 0.62	0.60 ± 0.47	0.61 ± 0.50
Me’	0.78 ± 0.83	0.63 ± 0.56	0.64 ± 0.57	0.69 ± 0.67
C	0.93 ± 0.45	0.87 ± 0.76	0.82 ± 0.92	0.87 ± 0.74
Ba	0.81 ± 0.84	0.96 ± 0.97	0.82 ± 0.67	0.86 ± 0.84
S	0.73 ± 0.45	0.48 ± 0.34	0.67 ± 0.42	0.63 ± 0.42
N’	1.11 ± 0.70	0.80 ± 0.69	1.12 ± 0.63	1.01 ± 0.69
P	0.92 ± 0.59	1.48 ± 1.39	0.97 ± 0.67	1.12 ± 0.98
Or	1.05 ± 0.77	0.70 ± 0.48	0.99 ± 0.65	0.91 ± 0.66
ANS	2.06 ± 1.32	0.90 ± 0.99	2.16 ± 1.15	1.71 ± 1.29
PNS	0.65 ± 0.36	0.67 ± 0.52	0.66 ± 0.47	0.66 ± 0.46
Pt	1.42 ± 1.03	1.49 ± 1.24	1.15 ± 1.27	1.35 ± 1.19
Ptm	0.91 ± 0.86	0.64 ± 0.43	0.85 ± 1.32	0.80 ± 0.95
A	1.70 ± 0.93	1.06 ± 0.72	1.30 ± 0.59	1.35 ± 0.81
U1	0.82 ± 0.58	0.50 ± 0.70	1.00 ± 0.66	0.77 ± 0.68
UIA	2.39 ± 1.05	0.80 ± 0.48	1.31 ± 0.82	1.50 ± 1.05
C1	1.17 ± 0.67	0.74 ± 0.79	0.61 ± 0.41	0.84 ± 0.69
LIA	1.60 ± 0.81	0.85 ± 0.94	0.73 ± 0.53	1.06 ± 0.87
B	1.34 ± 0.65	0.70 ± 0.68	0.96 ± 0.54	1.00 ± 0.68
Pog	0.81 ± 0.47	0.57 ± 0.56	0.50 ± 0.28	0.63 ± 0.44
Gn	0.80 ± 0.74	0.50 ± 0.60	0.43 ± 0.29	0.57 ± 0.60
Me	0.79 ± 0.62	0.62 ± 0.56	0.47 ± 0.28	0.63 ± 0.53
Go	1.87 ± 1.38	1.20 ± 0.96	1.04 ± 1.71	1.37 ± 1.43
Ar	0.95 ± 0.63	1.19 ± 0.87	0.86 ± 0.54	1.00 ± 0.71
Pcd	1.04 ± 0.91	1.09 ± 0.93	0.97 ± 0.45	1.03 ± 0.80
Co	1.18 ± 0.80	1.10 ± 0.67	1.38 ± 0.74	1.22 ± 0.75
Average	1.11 ± 0.48	0.80 ± 0.26	0.97 ± 0.51	/

In the Angelalign dataset, the glabella (G) exhibited the lowest MRE (0.46 ± 0.54 mm), while the porion (P) exhibited the highest MRE (1.48 ± 1.39 mm); in the MyOrthoX dataset, the pogonion of soft tissue (Pog’) exhibited the lowest MRE (0.61 ± 0.35 mm), while the root apex of upper central incisor (UIA) exhibited the highest MRE (2.39 ± 1.05 mm); in Digident dataset, the labrale inferius (Li) exhibited the lowest MRE (0.56 ± 0.29 mm), while pronasale (Prn) exhibited the highest MRE (2.43 ± 1.57 mm).

#### SDR

In the Angelalign dataset, the detection of gnathion (Gn) exhibited the highest SDR, while the pterygoid (Pt) exhibited the lowest SDR. In the MyOrthoX dataset, the subnasale (Sn) exhibited the highest SDR, while the root apex of the upper central incisor (UIA) exhibited the lowest SDR. In the Digident dataset, pronasale (Prn) exhibited the highest SDR, and pogoion (Pog) exhibited the lowest SDR (Table 3).

**Table 3:** Landmark detection results in terms of successful detection rate (SDR) within 1.0, 1. and 1.5 mm for each software of cephalometric analysis

Landmark	SDR								
	MyOrthoX			Angelalign			Digident		
	< 1.0 mm	< 1.5 mm	< 2.0 mm	< 1.0 mm	< 1.5 mm	< 2.0 mm	< 1.0 mm	< 1.5 mm	< 2.0 mm
G	0.84	0.95	1.00	0.95	0.95	0.95	0.93	1.00	0.98
Prn	0.60	0.95	0.98	0.95	0.95	0.95	0.19	0.37	0.47
Sn	0.91	0.98	1.00	0.88	0.98	0.98	0.86	0.93	0.98
Ls	0.70	0.88	0.98	0.84	0.98	0.98	0.91	0.98	1.00
Li	0.60	0.88	0.95	0.88	0.95	0.98	0.93	1.00	1.00
Si	0.42	0.63	0.88	0.81	0.95	0.98	0.53	0.74	0.91
Pog’	0.88	1.00	1.00	0.95	0.98	0.98	0.84	0.95	1.00
Gn’	0.74	1.00	1.00	0.95	0.95	0.98	0.88	0.98	0.98
Me’	0.84	0.95	0.98	0.91	0.98	0.98	0.84	0.93	0.98
C	0.56	0.93	0.98	0.67	0.91	0.93	0.81	0.91	0.93
Ba	0.72	0.84	0.95	0.72	0.81	0.91	0.67	0.86	0.91
S	0.79	0.93	1.00	0.95	0.98	0.98	0.77	0.98	1.00
N’	0.53	0.79	0.91	0.74	0.88	0.95	0.47	0.81	0.88
P	0.60	0.88	0.93	0.47	0.72	0.79	0.58	0.77	0.95
Or	0.63	0.81	0.88	0.86	0.91	0.95	0.65	0.88	0.93
ANS	0.28	0.35	0.49	0.72	0.84	0.91	0.21	0.26	0.35
PNS	0.84	0.98	1.00	0.79	0.91	0.93	0.77	0.93	1.00
Pt	0.47	0.63	0.81	0.40	0.67	0.79	0.56	0.81	0.86
Ptm	0.77	0.88	0.91	0.88	0.93	0.98	0.86	0.95	0.95
A	0.21	0.47	0.65	0.49	0.81	0.91	0.37	0.63	0.84
U1	0.58	0.93	0.98	0.93	0.95	0.98	0.51	0.84	0.95
UIA	0.05	0.26	0.35	0.70	0.93	0.95	0.42	0.65	0.74
C1	0.47	0.70	0.84	0.88	0.95	0.95	0.84	0.95	0.98
LIA	0.26	0.42	0.65	0.72	0.86	0.91	0.77	0.88	0.95
B	0.30	0.67	0.86	0.88	0.95	0.95	0.47	0.81	0.98
Pog	0.67	0.95	1.00	0.93	0.98	0.98	0.95	1.00	1.00
Gn	0.81	0.98	0.98	0.98	0.98	0.98	0.93	1.00	1.00
Me	0.79	0.93	0.95	0.84	0.98	0.98	0.95	0.98	1.00
Go	0.37	0.47	0.63	0.56	0.67	0.81	0.74	0.88	0.93
Ar	0.58	0.86	0.95	0.53	0.74	0.84	0.72	0.88	0.93
Pcd	0.67	0.74	0.88	0.63	0.77	0.86	0.60	0.84	1.00
Co	0.47	0.74	0.88	0.58	0.81	0.88	0.35	0.63	0.86

**Fig. 5:**
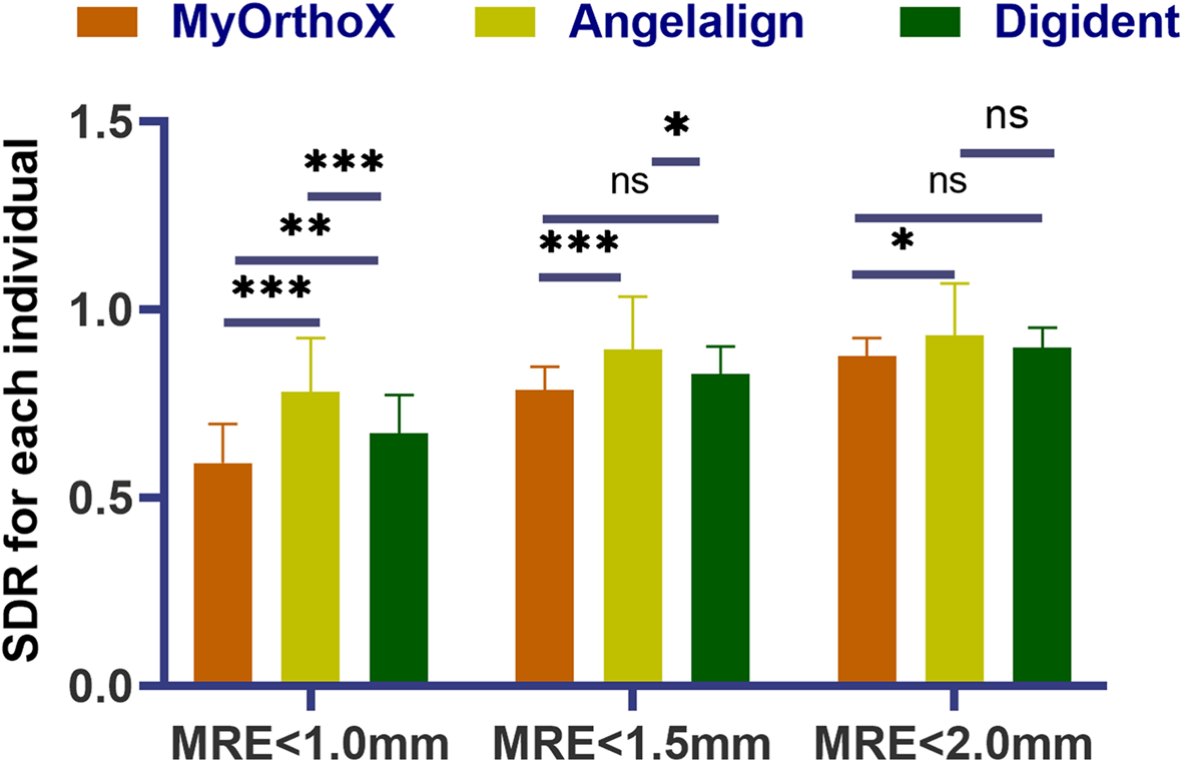
Comparison among the three AI-assisted groups in terms of the successful detection rate (SDR). One-way ANOVA analysis was applied to compare the average SDR among the groups within 1.0, 1.5, and 2.0 mm thresholds. Statistical significance was set at a *p*-value < 0.05

As for each individual, the Angelalign group had the highest average SDR, followed by the MyOrthoX and Digident groups (Fig. 5). Within 1.0, 1.5, and 2.0 mm threshold, the Angelalign group achieved average SDRs of 78.08%, 89.29%, and 93.09%, respectively, while the MyOrthoX group exhibited average SDRs of 67.02%, 82.80%, 89.99%, and the Digident group of 59.13%, 78.72%, 87.53%, respectively (Table 4).

**Table 4:** Successful detection rate (SDR) for each software of cephalometric analysis according to an independent individual

AI group	Classification	SDR ± SD (%)
MyOrthoX	MRE < 1.0 mm	67.02 ± 10.23
	MRE < 1.5 mm	82.80 ± 7.36
	MRE < 2.0 mm	89.99 ± 5.17
Angelalign	MRE < 1.0 mm	78.08 ± 14.23
	MRE < 1.5 mm	89.29 ± 14.02
	MRE < 2.0 mm	93.09 ± 13.64
Digident	MRE < 1.0 mm	59.13 ± 10.36
	MRE < 1.5 mm	78.72 ± 5.97
	MRE < 2.0 mm	87.53 ± 4.84

### Average time needed for cephalometric analysis

**Fig. 6:**
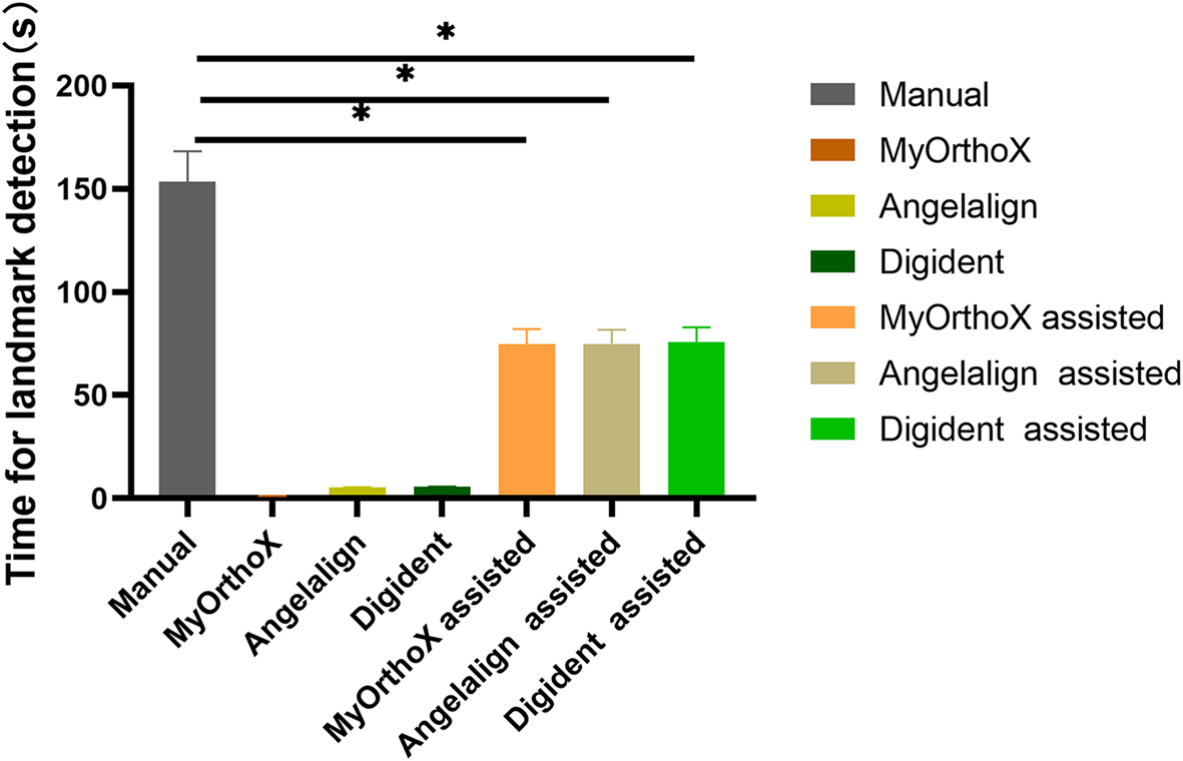
Mean time needed for landmark detection for the manual group, AI group, and AI-assisted group. Paired t-test was conducted to compare the average time for cephalometric analysis between AI-assistant groups (MyOrthoX-assisted, Angelalign-assisted, and Digident-assisted) and manual groups. Statistical significance was set at a *p*-value < 0.05

The average time and standard deviation of the time needed for each procedure of traditional cephalometric analysis were reported in seconds (Table 5). The AI-assisted group of commercial cephalometric analysis software needed less time than the manual group to perform cephalometric tracing and landmark location. The standard deviation of the mean time needed for tracing in the manual group was more than the AI-assisted group and the automated detection group (Fig. 6), indicating the wider range of individual variation in landmark tracing.

**Table 5:** Mean time needed for each software of cephalometric analysis

Group	Mean time ± SD (s)
Manual group	153.47 ± 14.83
AI group	
MyOrthoX	1.08 ± 0.12
Angelalign	5.18 ± 0.19
Digident	5.60 ± 0.20
AI-assisted group	
MyOrthoX-assisted	74.72 ± 7.31
Angelalign-assisted	74.98 ± 6.80
Digident-assisted	75.93 ± 6.91

### Impact of AI-assisted cephalometric landmarks detection on two aspects

#### Landmarks located on hard tissue

For common reference planes, the sella-nasion (SN) plane and cranial base (X-axis) were evaluated. Data in the Table 2 did not show a significant difference in MREs of nasion, orbitale (Or) and sella (S) among automated detection groups. As for landmarks located on soft tissue, the palatal plane was considered. The MRE of PNS also did not differ significantly among groups. However, the MRE of ANS in the Angelalign group exhibited the lowest, which denoted a more precise location of the palatal plane.

For the location of maxilla, pterygomaxillary fissure (Ptm) was evaluated. No significant difference was identified among the three groups, displaying an average MRE of 0.80 ± 0.95 mm and an average SDR of 93%. For the location of mandibular, landmarks involving condyle (Ar, Pcd, Co) and mentum (Pog, Gn, Me) were evaluated. In the digident group, the Ar and Pcd had the highest SDR (93% and 100%, respectively), while the Co exhibited the lowest SDR (86%). The Digident group achieved SDRs of 100% in landmarks associated with mentum within the 2 mm threshold.

Regarding the dentition, the incisal edge, and root apex were evaluated. In the Angelalign group, the U1and UIA exhibited the highest SDR within the 2 mm threshold (98% and 95%, respectively), while in the digident group, the lower incisor index C1 and LIA exhibited the highest SDR within the 2 mm threshold (98% and 95%, respectively).

### Landmarks located on soft tissue

As for landmarks located on soft tissue, the vertical reference line and nasolabial line were evaluated in this study, as well as the soft-tissue facial plane, esthetic line, T-line, and H-line. For the soft-tissue facial plane, MREs and SDRs related to pogonion of soft tissue (Pog’) and glabella (G) displayed no significance among groups, for an average MRE 0.52 ± 0.46 mm and 0.60 ± 0.51, respectively. For the esthetic line, the SDR of pronasale (Prn) was significantly higher in MyOrthoX and Angelalign groups than in the Digident group (47%). For the index of facial profile, the T-line consists of subnasale (Sn) and Pog’ while the H-line contains labrale superius (Ls) and Pog’. As shown in Table 2, no statistically significant differences in MREs were found among groups. For landmarks around mentum, Pog’ and gnathion of soft tissue (Gn’) did not differ significantly, however, menton of soft tissue (Me’) in the MyOrthoX group exhibited the highest MRE (0.79 ± 0.62 mm) among the automated detection groups,

## Discussion

The present study aimed to investigate which kind of the latest AI-assisted landmark detection programs would produce the most accurate results compared with experienced orthodontists. We selected three widely-used programs using machine learning methods to evaluate the strengths and weaknesses and reviewed the percentage of success in localizing each point. The MRE of the MyOrthoX, Angelalign, and Digident groups compared with the orthodontist group were 1.11 ± 0.48 mm, 0.80 ± 0.26 mm, and 0.97 ± 0.51 mm, respectively. Additionally, the SDR of MyOrthoX, Angelalign, and Digident within 2 mm of accuracy was 89.99%, 93.09%, and 87.53%, which is considered clinically acceptable. We tentatively proposed that the AI-assisted program can be considered a viable option for the repetitive and arduous task of landmark detection. The results of our study are consistent with the existing literature in terms of AI accuracy when identifying cephalometric landmarks within 2 mm [11, 20, 21].

The automated landmark detection task is a mix of structure detection, recognition, and estimation, including retrieving the relevant lines and exact shapes. The first attempt toward the automated landmark location on radiographs was made by Levy-Mandel. In their algorithm, the process was divided into transforming the line enhancement, line extraction, and landmark location [20, 22]. Rapidly evolving algorithms and increasing computational capabilities provide improved accuracy, reliability, and efficiency. Currently, methods of automatic cephalometric landmark detection are mainly separated into two major categories: bottom-up methods and learning-based methods [23]. Significant progress has been made in automatic landmark detection in cephalometry by using supervised machine-learning approaches. Zeng et al. developed a tree model to characterize the spatial layout patterns of facial landmarks for capturing facial structure information [24]. Ghesu et al. proposed a new paradigm by reformulating the detection problem and improving the detection speed of the reference methods by 2-3 orders of magnitude [25].

Although the automated detection program has achieved significant performance, there is still room for improvement in future work. Errors in cephalometric analysis include tracing, landmark identification, and measurement errors [26]. As for automated landmark identification, variations on individual skeletal structures, blurry images caused by device-specific projection magnifications, and image complexity due to overlapping contralateral structures remain to be solved [27]. Even a slight error can potentially cause misclassification that can lead to misdiagnosis, thus in our studies, we analyze the landmarks that are difficult to identify and prone to errors.

Among the hard tissue landmarks, MREs for few landmarks, including landmark 17 (posterior nasal spine), 23 (lower incisor), 30 (articular), 19 (pterygomaxillary fissure), and 21 (upper incisor), are especially high even for the best performing method [28]. These landmarks are difficult to identify precisely due to image complexity caused by the difference in X-ray projection between the left and right sides of the head structure [29]. As for the index of upper and lower incisors, open root apexes and malocclusion with dental crowding occasionally exist in patients with malocclusion, thus diminishing the accuracy of AI detection. The results of the studies conducted by Duran et al. using another automatic cephalometric analysis software (OrthoDx™ and WebCeph) also support these data [30]. When considering the reason that, condyle, gonion, and articular could be marked on two mandibular angle contours due to the limitation of the 2D lateral cephalogram. The identification of gonion-related angle and dentoalveolar height indicative of facial divergence in teenagers can help improve the capacity of clinicians to diagnose and treat participants. However, confusion can come from the fact that gonion is usually regarded as an average between two mandibular angle contours. Basion and orbitale are generally considered hard to detect and unreliable points in the cephalometric analysis [31]. The porion is also hard to locate because many similar radiolucencies resemble the radiolucency of the internal auditory meatus that exist in the search region. However, in our study, the average MREs of Basion, orbitale, and porion were 0.86 ± 0.84 mm, 0.91 ± 0.66 mm, and 1.12 ± 0.98 mm, which is considered clinically acceptable.

Among the soft tissue landmarks, landmark 2 (pronasale), 3 (subnasale), and 7 (pogonion of soft tissue) showed low SDRs due to higher darkness or lower brightness in these regions than the others [29, 32]. Problems with image quality influenced the ability of orthodontists who lacked experience in cephalometric landmark detection [33].

The time required to identify and trace the anatomical structures was also measured in our study. The orthodontist spends on average 15 min per analysis which is related to the quality of the cephalogram, the experience, and the number of points. A fully automated software would detect landmarks with fewer errors due to expert subjectivity, thus reducing the time required for analysis. As shown in Table 5, it takes two-fold less time in AI-assisted landmark detection than in manual groups. Once the landmarks are chosen on the digital images and identified, the data processing can be executed and completed immediately [34]. No significant difference in time consumption was observed among MyOrthoX, Angelalign, and Digident groups. There was no unequivocal trend that one modality in the benefit of saving time was always the best. However, the time required to make the digital measurements was substantially shorter than for the manual method, which is in line with the findings of other investigators.

The limitations of this study were the lack of evaluation of the consistency between orthodontists. It is noteworthy that even for experienced surgeons, significant variability exists when measuring radiographic parameters in patients. In a previous study that evaluated errors in cephalometric images, the researchers reported that inter-observer measurements showed a high correlation for both manual and digital measurements. The automated landmark detection software provides a process of automation through a knowledge-based machine learning algorithm. Machine learning algorithms learned directly from raw data without manual guidance, benefiting the discovery of the latent relationship. It has been reported that the presence of bias is one key challenge in the classifications and predictions of knowledge-based machine learning. In the literature reviews, it was seen that the bias of machine learning can be introduced to the decision-making process, including the human factor, poor quality of training data, model performance mismatch, and the infrastructure itself. Considering the variety of algorithms and landmark identification methods with the aid of artificial intelligence, more extensive further studies are needed.

To deploy AI responsibly, it is critical that algorithms used for prediction and diagnosis should be accurate and not lead to increased risk to patients. Our study determines whether automated landmark identification may perform better than orthodontic clinicians, and proposes that the latest cephalometry programs are capable to perform the analysis. Accepted ethical principles used to guide clinical research, must be prioritized and, in some cases, augmented [35]. Caution is necessary for the protection of personal data from ethical and legal viewpoints. In our retrospective study, the AI-assisted software used is free of charge on the websites and all methods were carried out by relevant guidelines and regulations. Clearly, there is still a need to integrate ethics into the development of AI algorithms, and more work is required to bridge the gap between AI in clinical diagnosis and treatment.

## Conclusion

The assistance of the AI in the assessment and retention of orthodontic treatment is an emerging area and the accuracy of the three commercially automatic landmark localizers (MyOrthoX, Angelalign, and Digident) was within acceptable ranges, which were capable of matching the reliability of experienced orthodontists. Additionally, AI-assisted software was of high efficiency and could potentially aid in clinical workflow and reduce research workload.

## Data Availability

The data underlying this article will be shared on reasonable request to the corresponding author.

## Abbreviations

MRE: Mean radical errors
SDR: Successful detection rate
AI: Artificial intelligence
